# Supplementary material for: IL-18 Signaling in the Rat Central Amygdala Is Disrupted in a Comorbid Model of Post-Traumatic Stress and Alcohol Use Disorder
Source: Cells. 2023 Jul 27;12(15):1943. doi: 10.3390/cells12151943 (PMC10416956; doi:10.3390/cells12151943)
Supplement: Supplementary file 1 [file cells-12-01943-s001.zip › cells-2506382-supplementary.pdf]

Supplementary materials

Title: IL-18 signaling in the rat central amygdala is disrupted in a comorbid model of post-traumatic stress and alcohol use disorder.

Authors:

Vittoria Borgonetti[1*], Bryan Cruz[1*], Valentina Vozella[1*], Sophia Khom[1,2], Michael Q. Steinman[1], Ryan Bullard[1], Shannon D'Ambrosio[1], Christopher S. Oleata[1], Roman Vlkolinsky[1], Michal Bajo[1], Eric P. Zorrilla[1], Dean Kirson[1,3] & Marisa Roberto[1#]

Affiliation:

[1]Department of Molecular Medicine, The Scripps Research Institute, La Jolla, CA, USA 92073

[2]Department of Pharmaceutical Sciences, University of Vienna, Josef-Holaubek-Platz 2, Vienna, A 1090, Austria

[2]Department of Pharmacology, Addiction Science, and Toxicology, The University of Tennessee Health Science Center, Memphis, TN, USA 38163

Correspondence:

* These authors contributed equally

[#] Marisa Roberto, The Scripps Research Institute, Department of Molecular Medicine, 10550 North Torrey Pines Road; La Jolla, California 92037; USA. Email: mroberto@scripps.edu

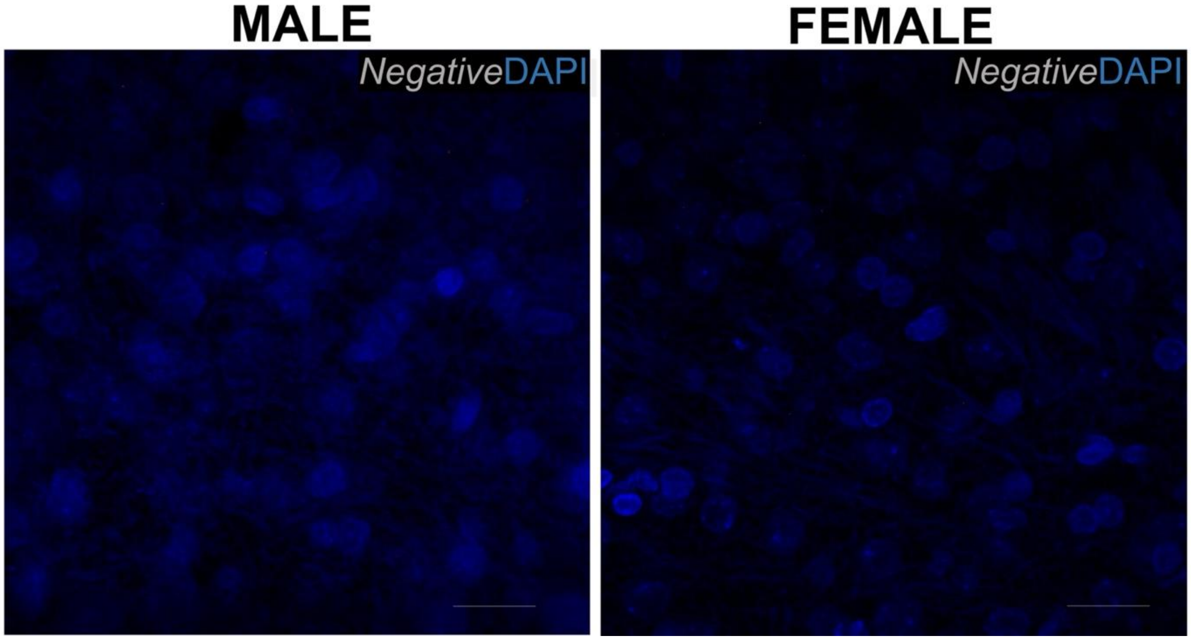

Supplementary Figure S1 Representative negative control images in the CeA of male and female indicate negligible fluorescence intensity for all the channels. Scale bar: 10 μm.
